# *tert*-Butyl 3-amino-2-methyl-6,7-dihydro-2*H*-pyrazolo[4,3-*c*]pyridine-5(4*H*)-carboxyl­ate

**DOI:** 10.1107/S1600536810013218

**Published:** 2010-04-17

**Authors:** Xin Guo, Xiao Guang Bai, Yi Liang Li, Yu Cheng Wang

**Affiliations:** a Institute of Medicinal Biotechnology, Chinese Academy of Medical Sciences, and Peking Union Medical College, Beijing 100050, People’s Republic of China

## Abstract

In the mol­ecule of the title compound, C_12_H_20_N_4_O_2_, the dihydro­piperidine ring assumes a half-chair conformation. In the crystal, cllassical N—H⋯O and N—H⋯N inter­molecular hydrogen bonds link mol­ecules into double chains along the *a* axis.

## Related literature

For the synthesis and properties of related kinase inhibitors, see: Fancelli *et al.* (2005 ▶); Gadekar *et al.* (1968 ▶).
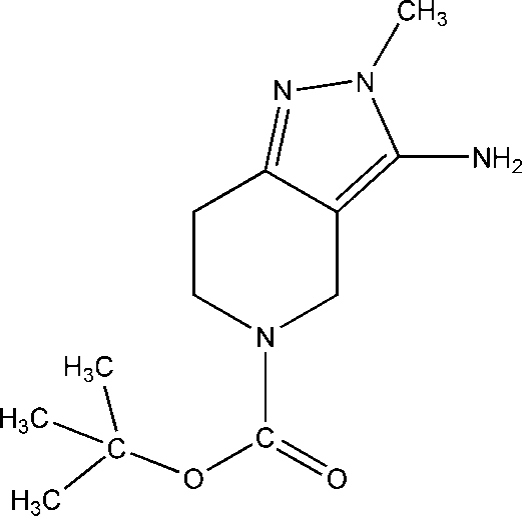


## Experimental

### 

#### Crystal data

C_12_H_20_N_4_O_2_
*M* *_r_* = 252.32
Triclinic, 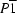

*a* = 6.3151 (13) Å
*b* = 9.3615 (19) Å
*c* = 11.215 (2) Å
α = 85.837 (4)°
β = 86.794 (4)°
γ = 87.733 (4)°
*V* = 659.8 (2) Å^3^
*Z* = 2
Mo *K*α radiation
μ = 0.09 mm^−1^
*T* = 293 K
0.30 × 0.26 × 0.16 mm

#### Data collection

Rigaku, SCXmini diffractometer
Absorption correction: multi-scan (*CrystalClear*; Rigaku, 2005 ▶) *T*_min_ = 0.972, *T*_max_ = 0.985
6859 measured reflections
3008 independent reflections
1737 reflections with *I* > 2σ(*I*)
*R*_int_ = 0.052

#### Refinement

*R*[*F* ^2^ > 2σ(*F* ^2^)] = 0.064
*wR*(*F* ^2^) = 0.170
*S* = 1.01
3008 reflections
167 parameters
H-atom parameters constrained
Δρ_max_ = 0.28 e Å^−3^
Δρ_min_ = −0.36 e Å^−3^

### 

Data collection: *CrystalClear* (Rigaku, 2005 ▶); cell refinement: *CrystalClear*; data reduction: *CrystalClear*; program(s) used to solve structure: *SHELXS97* (Sheldrick, 2008 ▶); program(s) used to refine structure: *SHELXL97* (Sheldrick, 2008 ▶); molecular graphics: *SHELXTL/PC* (Sheldrick, 2008 ▶); software used to prepare material for publication: *SHELXTL/PC*.

## Supplementary Material

Crystal structure: contains datablocks I, New_Global_Publ_Block. DOI: 10.1107/S1600536810013218/rz2434sup1.cif

Structure factors: contains datablocks I. DOI: 10.1107/S1600536810013218/rz2434Isup2.hkl

Additional supplementary materials:  crystallographic information; 3D view; checkCIF report

## Figures and Tables

**Table 1:** Hydrogen-bond geometry (Å, °)

*D*—H⋯*A*	*D*—H	H⋯*A*	*D*⋯*A*	*D*—H⋯*A*
N4—H4*A*⋯O2^i^	0.86	2.32	3.093 (3)	149
N4—H4*B*⋯N2^ii^	0.86	2.57	3.420 (3)	172

Symmetry codes: (i) 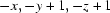; (ii) 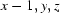.

**Fig. 1.**
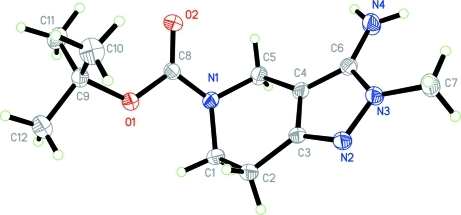
The molecular structure of the title compound, showing the atomic numbering scheme. Displacement ellipsoids are drawn at the 30% probability level.

**Fig. 2.**
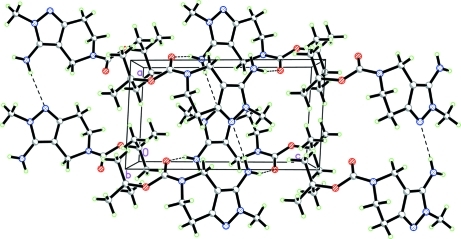
Packing diagram of the title compound viewed along the b axis. Intermolecular hydrogen bonds are shown as dashed lines.

## supplementary crystallographic information

### Comment

In our ongoing project aimed at the develpment of potential anticancer kinase inhibitors (Fancelli *et al.*, 2005; Gadekar *et al.*, 1968), we have synthesized the title compound and report its crystal structure herein.

In the molecule of the title compound (Fig. 1), bond lengths and angles are within the expected range. The dehydropiperidine ring assumes a half-chair conformation, with atoms N1 and C1 displaced from the C2–C5 mean plane by -0.4493 (19) and 0.293 (3)° respectively. In the crystal packing (Fig. 2), classical N—H···O and N—H···N intermolecular hydrogen bonds (Table 1) link molecules into double chains along the *a* axis.

### Experimental

A mixture of tert-butyl 3-cyano-4-oxopyrrolidine-1-carboxylate (2.1 g , 10.0 mmol) and methylhydrazine (0.46 g , 10.0 mol) was dissolved in ethanol (50 ml) and stirred at room temperature for 12 hours to give a white precipitate of the title compound. Colourless block crystals suitable for X-ray diffraction were obtained in 5 days by slow evaporation of a methanol solution (15 ml) of 100 mg of the crude product.

### Refinement

All H atoms were placed at calculated positions and refined as riding, with C—H = 0.96-0.97 Å, N—H = 0.86 Å, and with *U*_iso_(H) = 1.2*U*_eq_(C, N) or 1.5*U*_eq_(C) for methyl H atoms.

### Crystal data

**Table:**

C_12_H_20_N_4_O_2_	*Z* = 2
*M**_r_* = 252.32	*F*(000) = 272
Triclinic, *P*1	*D*_x_ = 1.270 Mg m^−^^3^
Hall symbol: -P 1	Mo *K*α radiation, λ = 0.71073 Å
*a* = 6.3151 (13) Å	Cell parameters from 5123 reflections
*b* = 9.3615 (19) Å	θ = 3.2–27.5°
*c* = 11.215 (2) Å	µ = 0.09 mm^−^^1^
α = 85.837 (4)°	*T* = 293 K
β = 86.794 (4)°	Block, colourless
γ = 87.733 (4)°	0.30 × 0.26 × 0.16 mm
*V* = 659.8 (2) Å^3^	

### Data collection

**Table:**

Rigaku, SCXmini diffractometer	3008 independent reflections
Radiation source: fine-focus sealed tube	1737 reflections with *I* > 2σ(*I*)
graphite	*R*_int_ = 0.052
Detector resolution: 13.6612 pixels mm^-1^	θ_max_ = 27.5°, θ_min_ = 3.2°
ω scans	*h* = −8→8
Absorption correction: multi-scan (*CrystalClear*; Rigaku, 2005)	*k* = −12→12
*T*_min_ = 0.972, *T*_max_ = 0.985	*l* = −14→14
6859 measured reflections	

### Refinement

**Table:**

Refinement on *F*^2^	Primary atom site location: structure-invariant direct methods
Least-squares matrix: full	Secondary atom site location: difference Fourier map
*R*[*F*^2^ > 2σ(*F*^2^)] = 0.064	Hydrogen site location: inferred from neighbouring sites
*wR*(*F*^2^) = 0.170	H-atom parameters constrained
*S* = 1.01	*w* = 1/[σ^2^(*F*_o_^2^) + (0.081*P*)^2^] where *P* = (*F*_o_^2^ + 2*F*_c_^2^)/3
3008 reflections	(Δ/σ)_max_ < 0.001
167 parameters	Δρ_max_ = 0.28 e Å^−^^3^
0 restraints	Δρ_min_ = −0.36 e Å^−^^3^

### Special details

**Table:**

Geometry. All esds (except the esd in the dihedral angle between two l.s. planes) are estimated using the full covariance matrix. The cell esds are taken into account individually in the estimation of esds in distances, angles and torsion angles; correlations between esds in cell parameters are only used when they are defined by crystal symmetry. An approximate (isotropic) treatment of cell esds is used for estimating esds involving l.s. planes.
Refinement. Refinement of *F*^2^ against ALL reflections. The weighted *R*-factor wR and goodness of fit *S* are based on *F*^2^, conventional *R*-factors *R* are based on *F*, with *F* set to zero for negative *F*^2^. The threshold expression of *F*^2^ > σ(*F*^2^) is used only for calculating *R*-factors(gt) etc. and is not relevant to the choice of reflections for refinement. *R*-factors based on *F*^2^ are statistically about twice as large as those based on *F*, and *R*- factors based on ALL data will be even larger.

### Fractional atomic coordinates and isotropic or equivalent isotropic displacement parameters (Å^2^)

**Table:**

	*x*	*y*	*z*	*U*_iso_*/*U*_eq_	
C1	0.3109 (4)	0.0363 (3)	0.6957 (2)	0.0386 (6)	
H1A	0.2825	−0.0408	0.6462	0.046*	
H1B	0.3436	−0.0061	0.7744	0.046*	
C2	0.5027 (4)	0.1173 (3)	0.6406 (2)	0.0398 (6)	
H2A	0.5619	0.1713	0.7007	0.048*	
H2B	0.6113	0.0498	0.6125	0.048*	
C3	0.4344 (4)	0.2180 (2)	0.5375 (2)	0.0331 (6)	
C4	0.2211 (4)	0.2453 (3)	0.5144 (2)	0.0329 (5)	
C5	0.0400 (4)	0.1836 (3)	0.59148 (19)	0.0385 (6)	
H5A	−0.0710	0.2567	0.6030	0.046*	
H5B	−0.0191	0.1057	0.5531	0.046*	
C6	0.2214 (4)	0.3437 (3)	0.4173 (2)	0.0346 (6)	
C7	0.5130 (4)	0.4687 (3)	0.2914 (2)	0.0479 (7)	
H7A	0.3984	0.5194	0.2522	0.072*	
H7B	0.5974	0.4162	0.2344	0.072*	
H7C	0.5997	0.5358	0.3251	0.072*	
C8	0.0740 (4)	0.2085 (3)	0.8027 (2)	0.0327 (5)	
C9	0.1596 (4)	0.2266 (3)	1.0122 (2)	0.0371 (6)	
C10	0.2487 (5)	0.3756 (3)	0.9930 (3)	0.0561 (8)	
H10A	0.3934	0.3684	0.9622	0.084*	
H10B	0.2431	0.4197	1.0679	0.084*	
H10C	0.1660	0.4329	0.9369	0.084*	
C11	−0.0678 (4)	0.2267 (3)	1.0624 (2)	0.0486 (7)	
H11A	−0.1532	0.2919	1.0136	0.073*	
H11B	−0.0739	0.2563	1.1427	0.073*	
H11C	−0.1206	0.1319	1.0627	0.073*	
C12	0.2991 (5)	0.1310 (3)	1.0927 (2)	0.0564 (8)	
H12A	0.2457	0.0360	1.1008	0.085*	
H12B	0.2984	0.1687	1.1701	0.085*	
H12C	0.4416	0.1279	1.0580	0.085*	
N1	0.1203 (3)	0.1310 (2)	0.70667 (17)	0.0353 (5)	
N2	0.5648 (3)	0.2928 (2)	0.46032 (17)	0.0388 (5)	
N3	0.4281 (3)	0.3700 (2)	0.38587 (17)	0.0386 (5)	
N4	0.0539 (3)	0.4037 (2)	0.35407 (19)	0.0507 (6)	
H4A	0.0781	0.4606	0.2915	0.061*	
H4B	−0.0744	0.3840	0.3773	0.061*	
O1	0.1814 (3)	0.15709 (17)	0.89818 (14)	0.0398 (4)	
O2	−0.0535 (3)	0.31087 (19)	0.80320 (15)	0.0480 (5)	

### Atomic displacement parameters (Å^2^)

**Table:**

	*U*^11^	*U*^22^	*U*^33^	*U*^12^	*U*^13^	*U*^23^
C1	0.0362 (14)	0.0381 (14)	0.0400 (14)	0.0063 (11)	0.0030 (11)	−0.0011 (11)
C2	0.0327 (14)	0.0447 (15)	0.0402 (14)	0.0078 (11)	0.0001 (11)	0.0032 (12)
C3	0.0273 (12)	0.0387 (14)	0.0327 (12)	0.0038 (10)	−0.0009 (10)	−0.0015 (11)
C4	0.0268 (12)	0.0436 (14)	0.0285 (12)	0.0024 (10)	−0.0019 (9)	−0.0044 (10)
C5	0.0308 (13)	0.0529 (16)	0.0319 (13)	−0.0022 (11)	−0.0021 (10)	−0.0040 (12)
C6	0.0296 (13)	0.0445 (14)	0.0299 (12)	0.0033 (11)	−0.0042 (10)	−0.0053 (11)
C7	0.0437 (16)	0.0557 (17)	0.0420 (15)	−0.0021 (13)	0.0000 (12)	0.0110 (13)
C8	0.0271 (12)	0.0374 (13)	0.0323 (12)	−0.0002 (11)	0.0005 (10)	0.0037 (11)
C9	0.0413 (15)	0.0385 (14)	0.0312 (13)	−0.0011 (11)	−0.0013 (10)	−0.0018 (11)
C10	0.0587 (19)	0.0517 (18)	0.0591 (18)	−0.0151 (14)	−0.0054 (14)	−0.0026 (14)
C11	0.0493 (17)	0.0514 (17)	0.0440 (15)	−0.0054 (13)	0.0103 (13)	−0.0028 (13)
C12	0.065 (2)	0.0602 (19)	0.0440 (16)	0.0080 (15)	−0.0120 (14)	0.0000 (14)
N1	0.0302 (11)	0.0431 (12)	0.0317 (10)	0.0020 (9)	0.0024 (8)	−0.0015 (9)
N2	0.0274 (11)	0.0496 (13)	0.0376 (11)	0.0047 (9)	−0.0007 (9)	0.0052 (10)
N3	0.0292 (11)	0.0487 (13)	0.0360 (11)	0.0037 (9)	−0.0020 (9)	0.0067 (10)
N4	0.0365 (13)	0.0750 (17)	0.0386 (12)	0.0059 (11)	−0.0059 (10)	0.0092 (11)
O1	0.0475 (11)	0.0406 (10)	0.0307 (9)	0.0096 (8)	−0.0045 (7)	−0.0031 (7)
O2	0.0519 (12)	0.0496 (11)	0.0403 (10)	0.0201 (9)	−0.0040 (8)	0.0006 (8)

### Geometric parameters (Å, °)

**Table:**

C1—N1	1.471 (3)	C8—O2	1.227 (3)
C1—C2	1.532 (3)	C8—O1	1.349 (3)
C1—H1A	0.9700	C8—N1	1.354 (3)
C1—H1B	0.9700	C9—O1	1.474 (3)
C2—C3	1.509 (3)	C9—C11	1.513 (3)
C2—H2A	0.9700	C9—C12	1.516 (3)
C2—H2B	0.9700	C9—C10	1.521 (3)
C3—N2	1.340 (3)	C10—H10A	0.9600
C3—C4	1.396 (3)	C10—H10B	0.9600
C4—C6	1.375 (3)	C10—H10C	0.9600
C4—C5	1.502 (3)	C11—H11A	0.9600
C5—N1	1.460 (3)	C11—H11B	0.9600
C5—H5A	0.9700	C11—H11C	0.9600
C5—H5B	0.9700	C12—H12A	0.9600
C6—N3	1.360 (3)	C12—H12B	0.9600
C6—N4	1.384 (3)	C12—H12C	0.9600
C7—N3	1.448 (3)	N2—N3	1.382 (3)
C7—H7A	0.9600	N4—H4A	0.8600
C7—H7B	0.9600	N4—H4B	0.8600
C7—H7C	0.9600		
			
N1—C1—C2	111.82 (19)	O1—C9—C11	111.03 (19)
N1—C1—H1A	109.3	O1—C9—C12	102.7 (2)
C2—C1—H1A	109.3	C11—C9—C12	110.1 (2)
N1—C1—H1B	109.3	O1—C9—C10	108.8 (2)
C2—C1—H1B	109.3	C11—C9—C10	113.5 (2)
H1A—C1—H1B	107.9	C12—C9—C10	110.2 (2)
C3—C2—C1	109.5 (2)	C9—C10—H10A	109.5
C3—C2—H2A	109.8	C9—C10—H10B	109.5
C1—C2—H2A	109.8	H10A—C10—H10B	109.5
C3—C2—H2B	109.8	C9—C10—H10C	109.5
C1—C2—H2B	109.8	H10A—C10—H10C	109.5
H2A—C2—H2B	108.2	H10B—C10—H10C	109.5
N2—C3—C4	112.4 (2)	C9—C11—H11A	109.5
N2—C3—C2	125.5 (2)	C9—C11—H11B	109.5
C4—C3—C2	122.1 (2)	H11A—C11—H11B	109.5
C6—C4—C3	105.4 (2)	C9—C11—H11C	109.5
C6—C4—C5	130.6 (2)	H11A—C11—H11C	109.5
C3—C4—C5	123.8 (2)	H11B—C11—H11C	109.5
N1—C5—C4	108.28 (18)	C9—C12—H12A	109.5
N1—C5—H5A	110.0	C9—C12—H12B	109.5
C4—C5—H5A	110.0	H12A—C12—H12B	109.5
N1—C5—H5B	110.0	C9—C12—H12C	109.5
C4—C5—H5B	110.0	H12A—C12—H12C	109.5
H5A—C5—H5B	108.4	H12B—C12—H12C	109.5
N3—C6—C4	106.7 (2)	C8—N1—C5	118.53 (19)
N3—C6—N4	123.2 (2)	C8—N1—C1	122.99 (19)
C4—C6—N4	129.9 (2)	C5—N1—C1	113.39 (19)
N3—C7—H7A	109.5	C3—N2—N3	103.51 (18)
N3—C7—H7B	109.5	C6—N3—N2	111.98 (19)
H7A—C7—H7B	109.5	C6—N3—C7	128.4 (2)
N3—C7—H7C	109.5	N2—N3—C7	119.56 (19)
H7A—C7—H7C	109.5	C6—N4—H4A	120.0
H7B—C7—H7C	109.5	C6—N4—H4B	120.0
O2—C8—O1	124.3 (2)	H4A—N4—H4B	120.0
O2—C8—N1	124.0 (2)	C8—O1—C9	121.18 (18)
O1—C8—N1	111.59 (19)		
			
N1—C1—C2—C3	−42.3 (3)	C4—C5—N1—C8	104.8 (2)
C1—C2—C3—N2	−171.7 (2)	C4—C5—N1—C1	−50.8 (3)
C1—C2—C3—C4	9.9 (3)	C2—C1—N1—C8	−86.9 (3)
N2—C3—C4—C6	−0.3 (3)	C2—C1—N1—C5	67.4 (2)
C2—C3—C4—C6	178.3 (2)	C4—C3—N2—N3	0.0 (3)
N2—C3—C4—C5	−175.9 (2)	C2—C3—N2—N3	−178.6 (2)
C2—C3—C4—C5	2.8 (4)	C4—C6—N3—N2	−0.7 (3)
C6—C4—C5—N1	−157.6 (2)	N4—C6—N3—N2	−177.5 (2)
C3—C4—C5—N1	16.7 (3)	C4—C6—N3—C7	−178.1 (2)
C3—C4—C6—N3	0.6 (3)	N4—C6—N3—C7	5.1 (4)
C5—C4—C6—N3	175.7 (2)	C3—N2—N3—C6	0.4 (2)
C3—C4—C6—N4	177.1 (2)	C3—N2—N3—C7	178.1 (2)
C5—C4—C6—N4	−7.8 (4)	O2—C8—O1—C9	−2.9 (3)
O2—C8—N1—C5	9.7 (3)	N1—C8—O1—C9	178.62 (19)
O1—C8—N1—C5	−171.83 (18)	C11—C9—O1—C8	60.3 (3)
O2—C8—N1—C1	162.8 (2)	C12—C9—O1—C8	177.9 (2)
O1—C8—N1—C1	−18.7 (3)	C10—C9—O1—C8	−65.3 (3)

### Hydrogen-bond geometry (Å, °)

**Table:**

*D*—H···*A*	*D*—H	H···*A*	*D*···*A*	*D*—H···*A*
N4—H4A···O2^i^	0.86	2.32	3.093 (3)	149
N4—H4B···N2^ii^	0.86	2.57	3.420 (3)	172

Symmetry codes: (i) −*x*, −*y*+1, −*z*+1; (ii) *x*−1, *y*, *z*.
